# Beyond Circulating Tumor Cell Enumeration: Cell-Based Liquid Biopsy to Assess Protein Biomarkers and Cancer Genomics Using the RareCyte^®^ Platform

**DOI:** 10.3389/fphar.2022.835727

**Published:** 2022-03-03

**Authors:** Eric P. Kaldjian, Arturo B. Ramirez, Lillian Costandy, Nolan G. Ericson, Walla I. Malkawi, Thaddeus C. George, Pashtoon Murtaza Kasi

**Affiliations:** ^1^ RareCyte, Inc, Seattle, WA, United States; ^2^ Department of Pharmaceutical Sciences and Experimental Therapeutics, College of Pharmacy, University of Iowa, Iowa City, IA, United States; ^3^ Division of Internal Medicine, Department of Hematology and Oncology, Weill Cornell Medicine, New York, NY, United States

**Keywords:** liquid biopsy, circulating tumor cell, protein biomarker, single cell sequencing, drug target, companion diagnostics, resistance phenotype, pharmacodynamics

## Abstract

The practice of medicine has steadily employed less invasive methods to obtain information derived from the tumor to guide clinical management of patients. Liquid biopsy—the sampling of blood—is a non-invasive method for generating information previously only available from tissue biopsies of the tumor mass. Analysis of fragmented circulating tumor DNA in the plasma is clinically used to identify actionable mutations and detect residual or recurrent disease. Plasma analysis cannot, however, assess cancer phenotypes, including the expression of drug targets and protein biomarkers. Circulating tumor cells (CTCs) are intact cancer cells that have entered the blood that have the potential for distant metastasis. While enumeration of CTCs is prognostic of outcome, recently developed technology allows for the interrogation of protein biomarkers on CTCs that could be predictive of response. Furthermore, since CTCs contain intact whole cancer genomes, isolating viable CTCs detected during therapy could provide a rational approach to assessing mutational profiles of resistance. Identification, characterization and molecular analysis of CTCs together will advance the capacity of liquid biopsy to meet the requirements of twenty-first century medicine.

## Introduction

Pathology is the discipline of medicine concerned with the analysis of tissues and body fluids to diagnose disease and so guide its treatment. Within oncologic pathology, there has been a steady historical trend in tumor tissue sampling and examination. Advances in methods and technologies have allowed access to tissues by diagnostic approaches that are increasingly less invasive to the patient: from tumor resection to excisional biopsy, incisional biopsy, core needle biopsy and then to fine needle aspiration biopsy. In the twenty-first century, precision treatments are directing therapy to the molecular drivers and differentiators of cancer rather than treatment based on the tissue of origin and morphologic subtype. Thus, increased amounts of molecular information at the protein expression (drug targets, biomarkers) and nucleic acid sequence (actionable mutations) level are demanded from biopsy samples even as the volume of the available material decreases.

After morphologic diagnosis, the biopsy sample may be used to identify phenotypic molecular sub-classifications that inform prognosis, to generate biomarkers of therapeutic response, and for companion diagnostic testing. Examples include estrogen, progesterone, and HER2 receptor testing in breast cancer to define hormonal or HER2 driven cancers; the use of the proliferation marker Ki-67 to characterize aggressiveness of cancer growth; and the testing of levels of PD-L1 expression that support treatment with immune checkpoint inhibitors. In addition, the biopsy sample is used for nucleic acid analysis at both the DNA and RNA level. Examples include sequencing of DNA for identification of mutations in receptors such as *EGFR* and *PIK3CA* to guide targeted therapies, and gene expression panel tests for determination whether chemotherapy should be included in the treatment of hormone receptor-positive breast cancer.

The least invasive way to access a tumor is by sampling blood—a “liquid biopsy”—rather than the tumor itself. In clinical practice this typically refers to circulating tumor DNA (ctDNA) based testing of plasma samples. However, the term broadly includes other elements of cancer that can be detected in the blood, including circulating tumor cells (CTCs). There are many advantages of liquid biopsy testing, including: 1) ease of access to samples; 2) minimal procedural discomfort and complications (considered non-invasive); 3) integration of tumor information from various locations throughout the body; 4) real-time data rather than historical snapshot; and 5) longitudinal sampling. Serial sampling allows for better assessment of intra-tumoral as well as temporal-heterogeneity in cancers, especially ones exposed to targeted therapies rather than chemotherapy (Burrell, et al., 2013). Analysis of ctDNA has proven to be very successful for detection of mutations that indicate susceptibility to targeted assays such as inhibitors of EGFR, as well as for monitoring the presence or recurrence of disease by assessment of mutational signatures. However, plasma analysis cannot generate the phenotypic data that tissue can provide, nor does it provide whole intact cancer genomes for interrogation. CTCs can rationally address these existing drawbacks inherent to ctDNA testing.

CTCs are intact cancer cells that have left the tissue compartment to enter the vascular compartment. There are two critical points regarding CTCs that are fundamental to the pathophysiology of tumor metastasis and progression, yet these are not widely recognized. The first is that *each distant tumor metastasis was once a CTC.* To spread to a distant site (not by local invasion or by the lymphatics) a cancer cell must enter the blood stream. Since it is estimated that more than 90% of cancer mortality is caused by distant metastasis, understanding and counteracting this process will be essential to managing cancer. Investigation of CTCs is likely to provide relevant information, since these cells are already “halfway” to metastasis: they have broken free from the tumor mass, transited through interstitial tissues, and intravasated into the vasculature. The second point is that *viable CTCs that are present during cancer therapy are resistant to treatment*. CTCs therefore represent a population of cells that can be investigated for mechanisms of resistance even if the therapy is successful in reducing tumor burden. These mechanisms may be phenotypic as well as genotypic. CTC liquid biopsy thus collects tumor cells with both metastatic and resistance potential. The incidence and number of detected CTCs is associated with cancer type and stage, increasing with more advanced disease. In metastatic cancers, the incidence of CTC-positive patients can be greater than 50%. Consequently, the use of CTC analysis in clinical research and practice is most appropriate for later stage disease (Vasseur, et al., 2021).

Nearly 2 decades ago, the CTC count was determined to be prognostic of outcome in breast cancer using the CellSearch system (Cristofanilli, et al., 2004). These results have been confirmed in many studies over the ensuing years, providing a substantial body of evidence that the presence and number of CTCs both before and after treatment reflects the aggressiveness of the cancer and its susceptibility to therapy. The clinical utility of CTC count determination, however, has not been definitively established, and hence it is currently more relevant to clinical research than to community practice. The reason for this is that while the CTC count is prognostic, it has not been demonstrated to be predictive: it can give an early indication that a therapy will not be effective (Lorente, et al., 2016), but it does not define the one that will be effective. This lack of positive decision-making power has left CTC enumeration as a test of tumor burden that supplies disease context to the physician, but not direction. To achieve clinical utility, CTCs must be able to provide the type of molecular information that is currently derived from tissue samples—protein biomarker and nucleic acid sequence data—and there is increasing focus on this (Keller and Pantel, 2019).

For analysis of both protein and nucleic acid biomarkers on individual CTCs, a technology platform that identifies them with high analytic sensitivity and specificity must be integrated with a method for assessment of the biomarkers on the cells confirmed as CTCs. The capacity to investigate more than one biomarker increases the depth of information about the cancer that may be used in clinical research. Furthermore, the ability to image at sub-cellular resolution enables localization of biomarkers to compartments (nucleus, cytoplasm, membrane) which can be important for understanding cell physiology. Finally, a method for isolation of individual cells is necessary. RareCyte has developed a platform and workflow that combines these features to support the next generation of cell-based liquid biopsy applications (Campton, et al., 2015; Kaldjian, et al., 2018).

### The RareCyte Liquid Biopsy Platform

The CTC analysis workflow includes four steps. The first three allow CTC enumeration and protein biomarker analysis: sample processing (AccuCyte^®^ system—blood collection, nucleated cell density separation, and slide preparation); immunofluorescence [IF] staining (RarePlex^®^ kits); and imaging (CyteFinder^®^ instruments—automated 7-channel scanning microscopes, analysis software). Briefly, the buffy coat fraction of the blood containing CTCs is separated and spread in a monolayer smear onto microscope slides; the slides are stained by IF using automated stainers; the stained slides are scanned, and image files are analyzed by automated software to define candidate CTCs for confirmation by a trained reviewer. Identification of epithelial CTCs requires three IF channels—1) cocktail of anti-pan-cytokeratin (CK) and anti-EpCAM antibodies; 2) anti-CD45 antibody; 3) nuclear dye. Two additional channels 4) and 5) are used for biomarkers that are included in validated assays or can be used for investigation novel markers using a kit that allows insertion of antibodies. A CTC must show staining in the CK/EpCAM channel, no staining in the CD45 channel, and must have a nucleus. Machine learning systems assist the reviewer by rank ordering candidate cells based on probability of being a CTC. CTC nucleic acid sequencing is accomplished in an additional final step in which identified CTCs are mechanically removed from the slide (CytePicker^®^ device, located above the stage in the CyteFinder instrument) and placed individually into tubes.

The RareCyte platform has been engineered to overcome inherent technology-driven biases in CTC detection (Castro-Giner and Aceto, 2020). For example, micro-fluidic devices using size exclusion to separate larger CTCs from white blood cells (WBC) do not collect small CTCs. Technologies that use positive selection by surface protein immuno-capture do not detect low antigen-expressing CTCs. Both types of technologies often require a second technology platform for protein biomarker imaging and CTC sequencing. The separation of nucleated cells from red cells and plasma based on differential density allows all CTCs in the blood sample to be collectively present for image-based identification. If detection of CTCs is considered a “needle in a haystack” problem, the RareCyte platform collects the haystack of nucleated cells without selection bias and then uses highly sensitive automated immunofluorescence microscopy to identify the “needles”.

By cocktailing surface EpCAM and cytoplasmic pan-cytokeratin markers together for identification of epithelial cells, the platform can identify CTCs by expression of either marker alone or in combination. This is particularly important in the identification of CTCs that lie within the continuum of the epithelial-mesenchymal transition (EMT). In EMT cells lose epithelial markers and take on mesenchymal ones, and it is increasingly understood that there are hybrid cells that express both phenotypes simultaneously (Pastushenko, et al., 2018); such E/M cells are thought to be critical to metastasis and are associated with poor outcomes (Deshmukh et al., 2021). It is possible that a CTC in EMT may lose EpCAM and other surface proteins and yet still retain cytokeratin within its cytoplasm that can be detected by the RareCyte CTC assay. A multi-marker CTC assay that incorporated EpCAM/cytokeratin and the mesenchymal marker vimentin has been used to identify E/M CTCs which were then retrieved and sequenced to demonstrate a specific mutation that confirmed prostate cancer origin (Ramirez, et al., 2018). Conclusive reports on the numbers of purely mesenchymal CTCs are lacking, since mesenchymal markers are shared with WBCs, making definitive identification difficult.

### Protein Biomarker Applications

#### Tumor characterization

Additional biomarker channels enable investigation of markers that may be known to be clinically significant within a tumor type, or of investigational interest. For instance, we have qualified and analytically validated CTC assays for androgen receptor (AR), AR splice variant 7, and synaptophysin (prostate cancer), HER2 and ER (breast cancer), and PD-L1 (immune-oncology). In addition, we have qualified additional markers, including PSMA, PSA, Muc1, progesterone receptor, EGFR, HER3 and Ki-67 for various epithelial cancers. These markers can be used in combination to define phenotypic subsets of cells. For instance, breast cancer CTCs can be divided into ER+/HER2+, ER+/HER2-, ER-/HER2+ and ER-/HER2-groups and their presence followed over time. Not all protein biomarker analyses would be of interest to all tumor types. The flexibility of the platform allows clinicians and investigators to choose the relevant markers of choice. For example, in patients with gastrointestinal (GI) malignancies, the hormone receptors have no role. However, other markers are of high interest, including EGFR and HER2, which has had intense drug development surrounding it over the past several years. The ability to assess this marker serially by blood sampling could prove highly useful, since tumors can gain or lose HER2 over time (Burrell, et al., 2013).

#### Diagnostic confirmation

There are clinical situations in which patients cannot undergo invasive diagnostic procedures because of risk, infrequent healthcare visits, cost or lack of access to advanced technology. In these settings, routine tissue biopsy procurement may be impossible. For example, some patients with a suspicious lung imaging lesion consistent with cancer cannot undergo bronchoscopy due to procedural risk; other patients with clinical symptoms consistent with cancer (such as long-term smoking exposure) may not have access to standard imaging for diagnostic evaluation; in global health settings invasive surgical procedures may not available. The detection of CTCs via a blood sample that includes confirmatory markers of tissue origin (EGFR in lung cancer or HER2 in breast cancer and GI malignancies, for instance) may establish a sound medical rationale for confirmation of a cancer diagnosis and subsequent treatment.

#### Companion diagnostics development

Drug targets and protein markers of therapeutic efficacy can be evaluated on actual tumor cells in the blood using CTC-based liquid biopsy. This provides a rational approach to the development of non-invasive companion diagnostics that are not sequence based.

#### Tumor transformation

Lineage differentiation biomarkers can be employed to ascertain the transformation of a tumor from one sub-type to another. For example, a significant fraction of castration resistant prostate cancers will transform to neuroendocrine (NE) prostate cancer which no longer responds to anti-androgen therapies. NE differentiation can be detected by expression of synaptophysin on CTCs, offering a rational minimally invasive alternative to procurement of tissue (typically bone marrow biopsy) for confirmation of transformation. Another type of transformation is epithelial-to-mesenchymal transition, which is understood to be an important aspect of cancer progression and metastasis. Current consensus is that cells are rarely purely mesenchymal, but typically retain some epithelial characteristics while gaining mesenchymal ones. By adding markers such as vimentin or N-cadherin to the epithelial CTC assay, transformation to mesenchymal phenotype can be followed.

#### Pharmacodynamic investigation

Understanding drug-target interaction at a molecular level via sequential tumor biopsies is exceedingly difficult in clinical studies. Yet it is extremely important to understand whether an investigational drug is having meaningful effect on the cells it is intended to treat. CTC biomarker assays make possible the assessment of drug activity via downstream markers known to be modulated by drug action.

#### Non-epithelial cancers

Most studies of CTCs are in populations of patients with epithelial tumors, that together constitute most cancers. First generation CTC technologies have used antibodies to epithelial surface markers (typically EpCAM, and others) to capture CTCs from the blood, which can then be confirmed by visualization of a second common epithelial marker, such as cytokeratin. This has been effective in identification of CTCs that express both markers, although CTCs that express only one will be missed using this approach. Not all cancers are epithelial, however (in fact the first reported case of circulating tumor cells by Ashworth in 1869 was in a patient with a sarcoma), and CTCs from non-epithelial cancers will not be detected using epithelial markers. This limitation can be overcome with a platform that allows flexible substitution of other markers for specific identification of the cell type. For example, RareCyte has qualified antibodies to S-100, melan-A, and NG2 for identification of circulating melanoma cells.

### Nucleic Acid Sequencing Applications

#### Targeted mutation analysis

Cell-free DNA is most effectively employed for targeted sequencing of circulating tumor DNA (ctDNA). However, ctDNA analysis is not always successful; allelic fraction and total amount of ctDNA must be sufficient for confident mutation detection. CTCs contain “pure” cancer genomes, minimizing if not eliminating the influence of allelic fraction on sensitivity. The RareCyte platform has been used to identify actionable PIK3CA mutations in CTCs that were not identified in plasma using the same targeted panel in patients with metastatic breast cancer (Liu, et al., 2020).

#### Whole genome/exome sequencing

CTCs contain the entire intact cancer genome, allowing genome-wide sequencing that can be employed for broad detection of mutations. The RareCyte platform has been used together with whole exome sequencing to follow the genomic evolution of CTCs in triple-negative breast cancer over the course of 9 months (Kaldjian, et al., 2018). Since methods for genome amplification continue to improve, this approach will likely become more common.

#### Breakthrough/resistant clone detection

Apoptotic cells are understood to release ctDNA that is detectable in the plasma in short fragments that can be tested for mutations. Resistant clones may comprise only a small fraction of the entire tumor mass, and they are less likely to undergo apoptosis, so their genomes may not be detected at the current limit of sensitivity of cell-free DNA assays. As discussed above, there is a rational basis for higher representation of resistant clones within the viable CTC population. When patients are undergoing therapy, CTCs present may be identified, isolated, and sequenced to find actionable mutations within the resistant population are not present in the bulk of the tumor to guide planning for subsequent treatment.

#### Tumor cell heterogeneity

ctDNA analysis can assess mutational heterogeneity across a bulk population of tumor cells but cannot resolve mutational profiles within individual cells. Heterogeneity is inferred by presence of different variant allele fractions of mutations, which often tend to be subclonal (Misale, et al., 2014). Since CTCs can be sequenced individually, they potentially better reflect the variability amongst clones that have acquired different mechanisms of resistance. Thus, CTCs are better positioned to study heterogeneity at the cellular level.

### Clinical Examples

#### CTC enumeration and biomarker analysis

**FIGURE 1:**
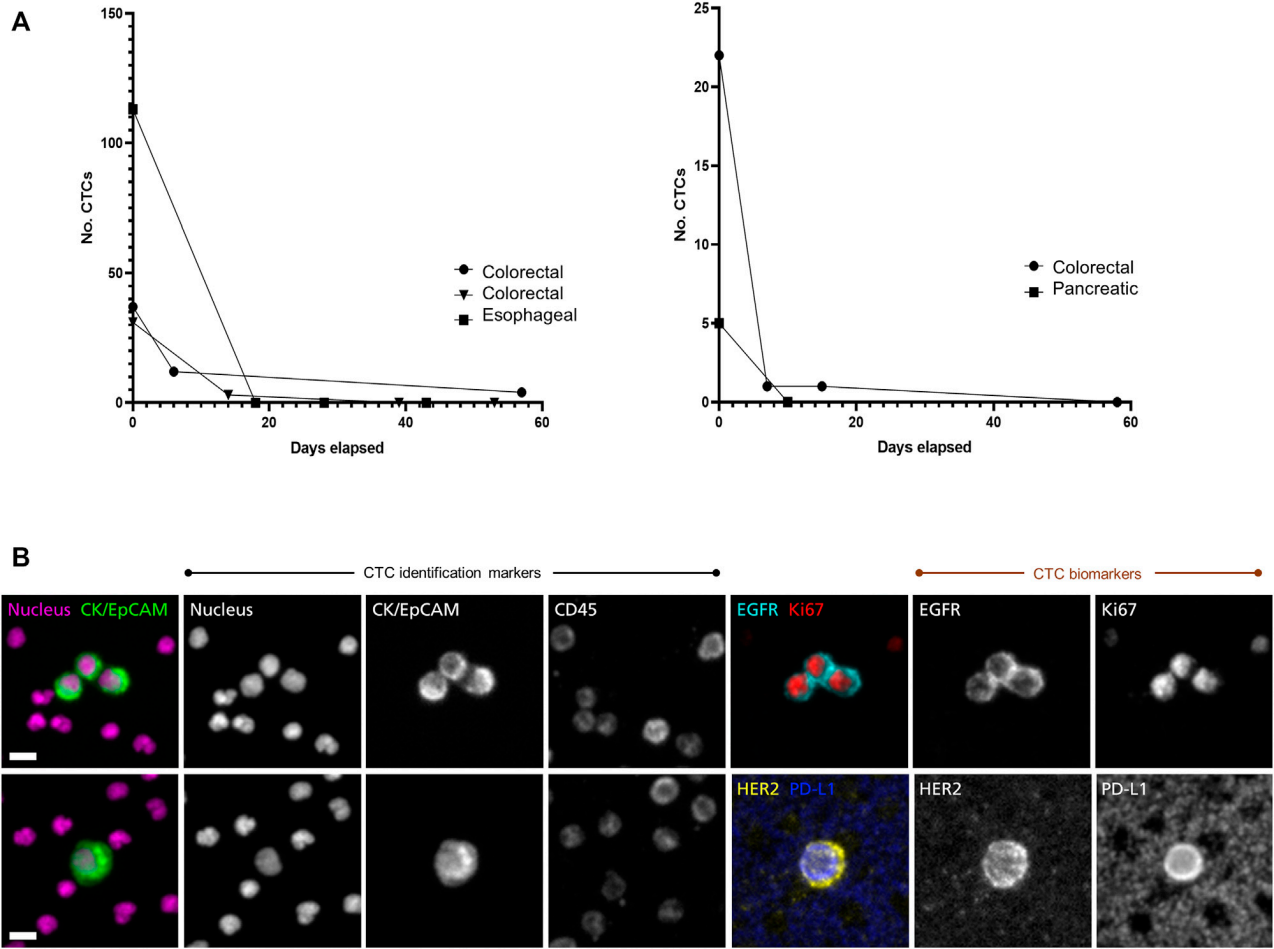
Longitudinal monitoring of CTC counts and characterization of CTC biomarkers. The RareCyte platform was used to study CTCs in a diverse set of gastro-intestinal cancer patients with advanced disease **(A)** CTC counts per 7.5 ml sample were enumerated pre-treatment and followed during treatment course. CTC counts dropped rapidly in several patients of various cancer types (colorectal, esophageal, pancreatic) that later showed clinical evidence of response to therapy by medical imaging and/or serum biomarkers (data not shown). This was seen in patients with high CTC counts (left panel) as well as in patients with lower CTC counts (right panel) **(B)** CTC assays used in the study included dual-biomarker assays, one characterizing EGFR and Ki-67 and a second characterizing HER2 and PD-L1. In each assay CTCs were identified by use of nuclear, epithelial (cytokeratin/EpCAM), and leukocyte (CD45) markers. Identified CTCs were then assessed for expression of the investigative biomarkers. The CTC images shown here are from a patient with progressing esophageal cancer. The trio of CTCs in the upper panel express EGFR on the cell membrane and Ki-67 in the nucleus, indicating that they are in proliferative phase. The CTC in the lower panel expresses HER2 as well as PD-L1. Note that background platelets also express PD-L1. Scale bar = 10 µm.

In a clinical study of patients with advanced gastrointestinal cancers, CTCs were counted and followed during treatment. Figure 1A shows rapid drops in CTC counts in responding patients. Using two dual-biomarker assays (EGFR/Ki-67 and HER2/PD-L1), four biomarkers were investigated. Figure 1B shows a cluster of CTCs positive for EGFR and Ki-67, and a single CTC positive for HER2 and PD-L1 from a patient with esophageal cancer.

#### Targeted panel sequencing of individual CTCs

**FIGURE 2:**
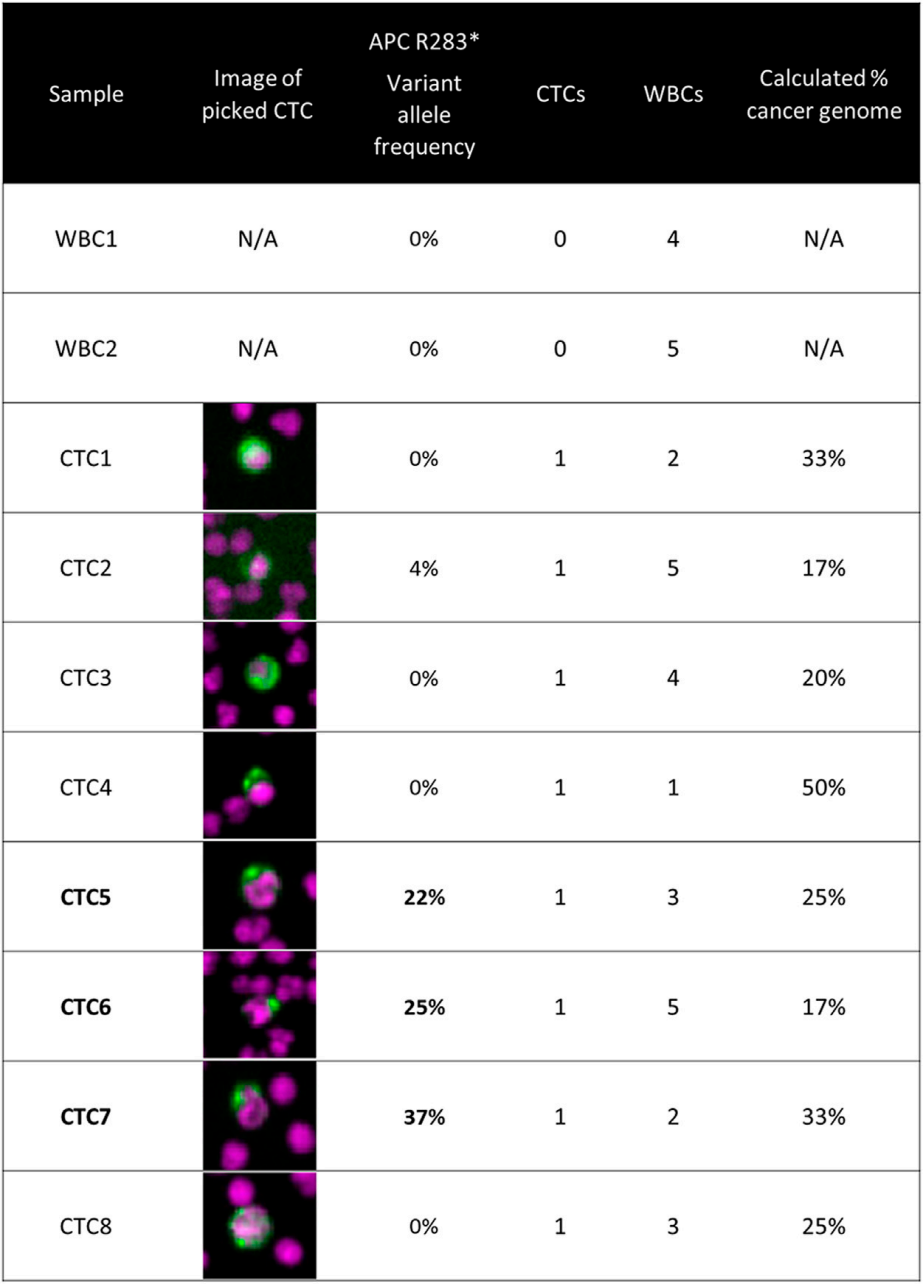
Targeted panel sequencing of individual CTCs from a colorectal cancer patient. Eight CTCs were sequenced using a 65-gene cancer hotspot panel. An *APC* nonsense mutation (R283*) was identified in three of the CTCs, with variant allele frequencies ranging from 22 to 37%. The image of the CTC retrieved for sequencing is displayed in the second column. The number co-retrieved white blood cells and the calculated cancer genome fraction after white blood cell dilution are indicated for each sample. Methods: Blood was processed using the RareCyte platform, stained with either of the dual biomarker assays described in Figure 1, and imaged. Visualized CTCs were retrieved from the microscope slides and deposited into PCR tubes. Cells were lysed and then library preparation was performed using CleanPlex^®^ OncoZoom^®^ Cancer Hotspot Panel (Paragon Genomics) with a modified protocol to sequence single cells. Resulting libraries were sequenced on an MiSeq System (Illumina) followed by alignment and variant calling. To be called as somatic mutations, variants had to be present in more than one CTC sample at a minimum variant allele frequency threshold of 10%, be absent in germline WBC controls, and be listed in COSMIC database v71.

CTCs from a colorectal cancer patient were retrieved from slides after imaging for hotspot sequencing. The presence of an APC nonsense mutation was detected in three of eight CTCs. This is described in detail in Figure 2.

## Discussion

The promise of liquid biopsy is that it will provide the types of valuable information for research and clinical care that currently require tissue biopsies. The many advantages of cell-based liquid biopsy have been discussed in the introduction, but there are limitations. Not all cancer patients have CTCs in the volume of blood typically used for analysis (<10 ml), especially in early clinical stage disease. Yet for many patients, especially those with advanced or recurrent disease, CTCs are not limiting, and thus are a source of cancer tissue for serial real-time analysis. It is important to use highly sensitive detection technologies that are integrated with analytic methods for protein and nucleic acid analysis.

Evaluation of cellular protein biomarkers in tissues is part of standard pathology laboratory practice. Many clinically important protein biomarkers cannot be assessed by sequence, and so require cancer cells for assessment rather than soluble biomolecules. Technologies and methods have been developed that robustly identify CTCs and can assay multiple biomarkers on them. This has the potential for broad impact in tumor characterization and diagnostic confirmation, companion diagnostics development, monitoring of tumor transformation, pharmacodynamic investigation, non-epithelial cancers, and minimal residual disease monitoring.

Sequence and gene expression analysis of tissue samples to identify cancer mutations and profiles is routinely performed by molecular pathology laboratories. The fact that CTCs have a complete cancer genome and transcriptome, are critical to cancer metastasis, and may be enriched for resistant clones, suggests that genomic and transcriptomic analysis of CTCs may provide useful information that is not available in ctDNA. Since plasma and CTCs can both be isolated from the same blood collection tube, a strategy of plasma sequencing reflexing to CTC sequencing in cases where the ctDNA result is non-informative is a rational approach to increasing diagnostic sensitivity. This workflow is consistent with the concept that liquid biopsy methods should not be considered mutually exclusive. Similar to how liquid biopsies have not replaced tissue biopsies, CTC testing is not intended to replace ctDNA-based testing. Rather, the different approaches are best understood as complementing one another to provide a more complete assessment of cancer.

We have described a variety of CTC protein biomarker and sequencing applications in research and diagnostics based on an established liquid biopsy technology platform. There are other specimen types that can be classified as “liquid biopsy” that are routinely processed in the cytology laboratory. These include body fluids (such as pleural, peritoneal and cerebrospinal fluids, urine) and fine needle aspirate biopsies in which cellular fluid is aspirated from a tumor. Slide smears or monolayer preparations are made for morphologic evaluation and molecular testing. These small-volume samples can be difficult to thoroughly evaluate by traditional methods that require multiple slides for immunohistochemistry or sufficient material for sequencing. But since they are similar to buffy coat smears, they could be analyzed using the liquid biopsy methods described here for multi-protein biomarkers and target cell sequencing (Zhu, et al., 2021).

The promise of liquid biopsy is that it will be able to do what tissue biopsy has done—generate essential information for the diagnosis, phenotyping and molecular analysis of cancer. The challenge for liquid biopsy is to combine appropriate technologies with robust assay methods that can extract that information from blood components. ctDNA sequencing has rapidly gained adoption as a mutational testing method. We have described here how the phenotypic and molecular analysis of CTCs can supplement ctDNA sequencing to advance the capacity of liquid biopsy to meet the requirements of twenty-first century medicine.

## Data Availability

The original contributions presented in the study are publicly available. This data can be found here: https://www.ncbi.nlm.nih.gov/sra/, PRJNA791405.

## References

[B1] Burrell R. A. McGranahan N. Bartek J. Swanton C. (2013). The Causes and Consequences of Genetic Heterogeneity in Cancer Evolution. Nature 501, 338–345. 10.1038/nature12625 24048066

[B2] Campton D. E. Ramirez A. B. Nordberg J. J. Drovetto N. Clein A. C. Varshavskaya P. (2015). High-recovery Visual Identification and Single-Cell Retrieval of Circulating Tumor Cells for Genomic Analysis Using a Dual-Technology Platform Integrated with Automated Immunofluorescence Staining. BMC Cancer 15, 360. 10.1186/s12885-015-1383-x 25944336 PMC4430903

[B3] Castro-Giner F. Aceto N. (2020). Tracking Cancer Progression: from Circulating Tumor Cells to Metastasis. Genome Med. 12, 31. 10.1186/s13073-020-00728-3 32192534 PMC7082968

[B4] Cristofanilli M. Budd G. T. Ellis M. J. Stopeck A. Matera J. Miller M. C. (2004). Circulating Tumor Cells, Disease Progression, and Survival in Metastatic Breast Cancer. N. Engl. J. Med. 351, 781–791. 10.1056/NEJMoa040766 15317891

[B5] Deshmukh A. P. Vasaikar S. V. Tomczak K. Tripathi S. den Hollander P. Arslan E. (2021). Identification of EMT Signaling Cross-Talk and Gene Regulatory Networks by Single-Cell RNA Sequencing. Proc. Natl. Acad. Sci. U S A. 118 (19), e2102050118. 10.1073/pnas.2102050118 33941680 PMC8126782

[B6] Kaldjian E. P. Ramirez A. B. Sun Y. Campton D. E. Werbin J. L. Varshavskaya P. (2018). The RareCyte^®^ Platform for Next-Generation Analysis of Circulating Tumor Cells. Cytometry 93, 1220–1225. 10.1002/cyto.a.23619 30277660 PMC6586054

[B7] Keller L. Pantel K. (2019). Unravelling Tumour Heterogeneity by Single-Cell Profiling of Circulating Tumour Cells. Nat. Rev. Cancer 19, 553–567. 10.1038/s41568-019-0180-2 31455893

[B8] Liu M. C. Giridhar K. V. Leon Ferre R. A. Carroll J. L. Goetz M. P. Haddad T. C. (2020). Comparison of Circulating Tumor Cell (CTC) Derived DNA and Circulating Cell-free DNA (cfDNA) from Simultaneous Blood Sampling of Patients with Metastatic Breast Cancer (MBC). Cancer Res. 80 (16 Suppl. l), 3119. 10.1158/1538-7445.AM2020-3119

[B9] Lorente D. Olmos D. Mateo J. Bianchini D. Seed G. Fleisher M. (2016). Decline in Circulating Tumor Cell Count and Treatment Outcome in Advanced Prostate Cancer. Eur. Urol. 70, 985–992. 10.1016/j.eururo.2016.05.023 27289566 PMC5568108

[B10] Misale S. Di Nicolantonio F. Sartore-Bianchi A. Siena S. Bardelli A. (2014). Resistance to Anti-EGFR Therapy in Colorectal Cancer: from Heterogeneity to Convergent Evolution. Cancer Discov. 4, 1269–1280. 10.1158/2159-8290.CD-14-0462 25293556

[B11] Pastushenko I. Brisebarre A. Sifrim A. Fioramonti M. Revenco T. Boumahdi S. (2018). Identification of the Tumour Transition States Occurring during EMT. Nature 556, 463–468. 10.1038/s41586-018-0040-3 29670281

[B12] Ramirez A. B. Ericson N. G. Campton D. E. Duplessis M. Mojica T. Clein A. C. (2018). Development of a Multi-Parameter Immunofluorescence Assay for Identification of Circulating Tumor Cells with Epithelial-Mesenchymal Phenotype. Cancer Res. 78 (13 Suppl. l), 5190. 10.1158/1538-7445.AM2018-5190

[B13] Vasseur A. Kiavue N. Bidard F. C. Pierga J. Y. Cabel L. (2021). Clinical Utility of Circulating Tumor Cells: an Update. Mol. Oncol. 15, 1647–1666. 10.1002/1878-0261.12869 33289351 PMC8169442

[B14] Zhu Y. Allard G. M. Ericson N. G. George T. C. Kunder C. A. Lowe A. C. (2021). Identification and Characterization of Effusion Tumor Cells (ETCs) from Remnant Pleural Effusion Specimens. Cancer Cytopathol 129, 893–906. 10.1002/cncy.22483 34171181

